# The Chicago Medical Examiner

**Published:** 1871-12

**Authors:** 


### The Chicago Medical Examiner.

We copy from this Journal the following:

EXPLANATION.—The present number of the EXAMINER was just ready for delivery, but had not been sent out of the printing office, which was located in the North Division of the city, when the great fire occurred. It was consequently destroyed so completely that not even a scrap of the original manuscript was left. Some of the articles were soon reproduced and others substituted with a view to a speedy re-publication. But the fire had destroyed every press in the city in which book and medical journal work could be printed. We have waited patiently five or six weeks in the hope that our old printers would be ready to resume work, but have at last been obliged to send to Ann Arbor for the printing of the present number. The November and December numbers will follow in quick succession, and by the first of January we expect to be on time with the first number of the new volume.

The subscription list and all the books belonging to the EXAMINER were preserved. We hope all subscribers who are in arrears will immediately pay up, and new ones send in their names for the next volume.

